# Supplementary material for: Splice-Junction-Based Mapping of Alternative Isoforms in the Human Proteome
Source: Cell Rep. Author manuscript; Available in PMC 2020 Jan 15. (PMC6961840; doi:10.1016/j.celrep.2019.11.026)
Supplement: 2 [file NIHMS1546469-supplement-2.zip › DF1/PXD006675/PulmonaryArtery/PulmonaryArtery_23_BBS1_EGQSAPLLSAHVNMPGSEGLAAPNRPLNPE.pdf]

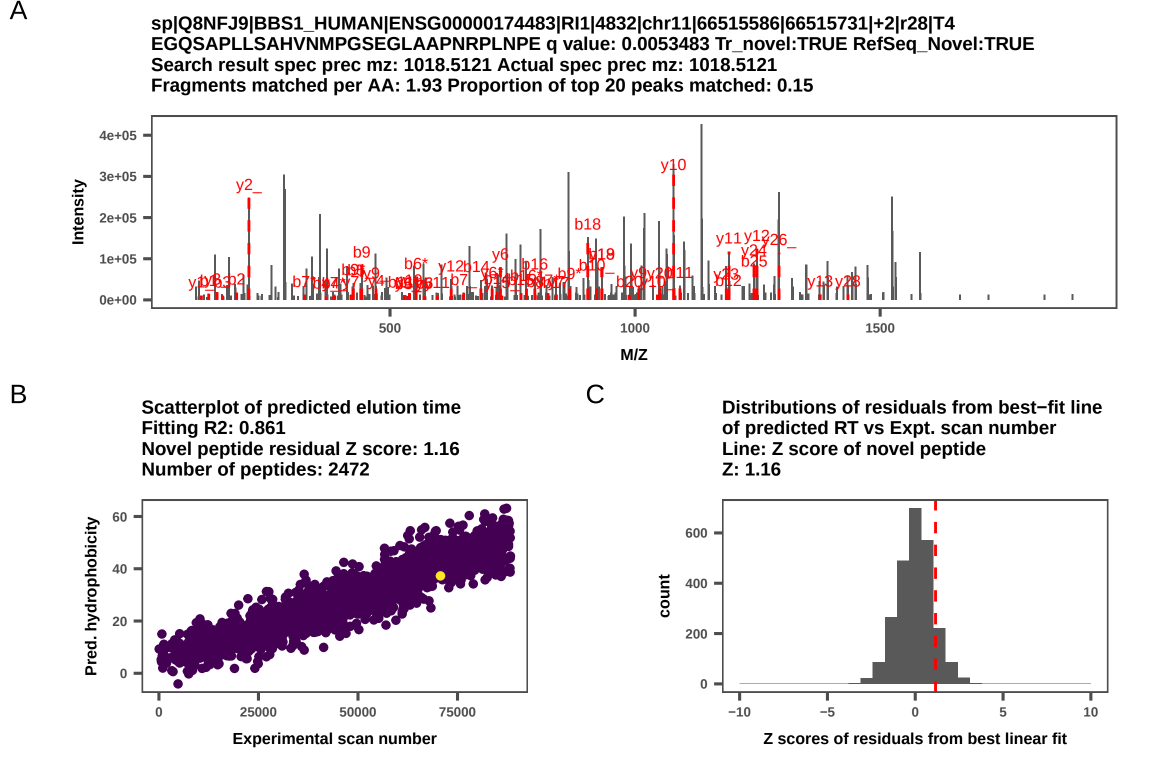
A
sp|Q8NFJ9|BBS1_HUMAN|ENSG00000174483|RI1|4832|chr11|66515586|66515731|+2|r28|T4
EGQSAPLLSAHVNMPGSEGLAAPNRPLNPE q value: 0.0053483 Tr_novel:TRUE RefSeq_Novel:TRUE
Search result spec prec mz: 1018.5121 Actual spec prec mz: 1018.5121
Fragments matched per AA: 1.93 Proportion of top 20 peaks matched: 0.15
Intensity
4e+05
3e+05
2e+05
1e+05
0e+00
y2_
y10
b18
b9
b6*
y6
b16
y11
y12
y26_
M/Z
500
1000
1500
B
Scatterplot of predicted elution time
Fitting R2: 0.861
Novel peptide residual Z score: 1.16
Number of peptides: 2472
Pred. hydrophobicity
60
40
20
0
0
25000
50000
75000
Experimental scan number
C
Distributions of residuals from best−fit line
of predicted RT vs Expt. scan number
Line: Z score of novel peptide
Z: 1.16
count
600
400
200
0
−10
−5
0
5
10
Z scores of residuals from best linear fit
